# Findings by an International Collaboration on SJS/TEN With Severe Ocular Complications

**DOI:** 10.3389/fmed.2021.649661

**Published:** 2021-12-01

**Authors:** Mayumi Ueta

**Affiliations:** Department of Ophthalmology, Kyoto Prefectural University of Medicine, Kyoto, Japan

**Keywords:** Stevens-Johnson syndrome (SJS), toxic epidermal necrolysis (TEN), severe ocular complications (SOC), HLA, cold medicine

## Abstract

Stevens-Johnson Syndrome (SJS) is an acute inflammatory vesiculobullous reaction of the skin and mucosa, e.g., the ocular surface, oral cavity, and genitals. In patients with extensive skin detachment and a poor prognosis, the condition is called toxic epidermal necrolysis (TEN). Not all, but some patients with SJS/TEN manifest severe ocular lesions. Approximately 50% of SJS/TEN patients diagnosed by dermatologists and in burn units suffer from severe ocular complications (SOC) such as severe conjunctivitis with pseudomembrane and ocular surface epithelial defects in the acute stage. In the chronic stage, this results in sequelae such as severe dry eye and visual disturbance. Before 2005, our group of Japanese scientists started focusing on ophthalmic SJS/TEN with SOC. We found that cold medicines were the main causative drugs of SJS/TEN with SOC and that in Japanese patients, HLA-A^*^02:06 and HLA-B^*^44:03 were significantly associated with cold medicine-related SJS/TEN with SOC (CM-SJS/TEN with SOC). We expanded our studies and joined scientists from Korea, Brazil, India, Taiwan, Thailand, and the United Kingdom in an international collaboration to detect the genetic predisposition for SJS/TEN with SOC. This collaboration suggested that in Japanese patients, cold medicines, including NSAIDs, were the main causative drugs, and that HLA-A^*^02:06 was implicated in Japanese and Korean patients and HLA-B^*^44:03 in Japanese-, Indian-, and European ancestry Brazilian patients. Our joint findings reveal that there are ethnic differences in the HLA types associated with SJS/TEN with SOC.

## Introduction

Stevens-Johnson syndrome (SJS) is an acute inflammatory vesiculobullous reaction of the mucosa of the ocular surface, oral cavity, and genitals, and of the skin. In patients with extensive skin detachment and a poor prognosis, the condition is called toxic epidermal necrolysis (TEN). In the acute stage of SJS/TEN, approximately 50% of patients present with severe ocular lesions such as severe conjunctivitis with pseudomembrane and ocular surface epithelial defects (1).

Ophthalmologists encounter patients not only in the acute- but also the chronic stage. Dermatologists, on the other hand, tend to see SJS/TEN patients only in the acute stage, although in some countries such as France and Germany dermatologists also followed up the patients long time. Our ophthalmologic diagnosis of SJS/TEN was based on a confirmed history of acute-onset high fever, serious mucocutaneous illness with skin eruptions, and involvement of at least two mucosal sites, including the ocular surface (2–9). SJS/TEN patients with severe ocular complications (SOC) in the acute stage often develop sequelae such as vision loss and very severe dry eye that prevent their having a normal life (10).

**Figure 1:**
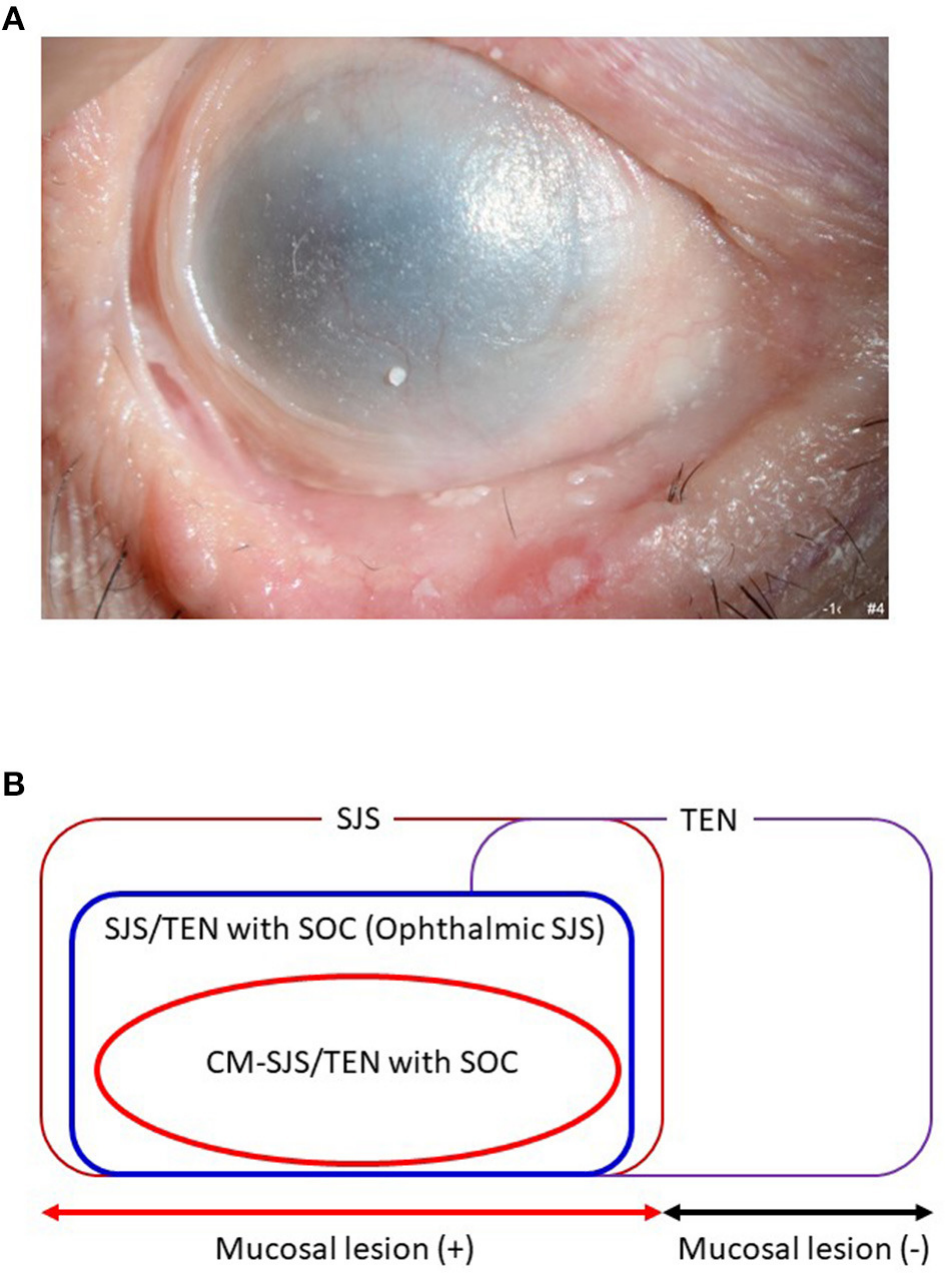
**(A)** Ocular surface finding of SJS/TEN with SOC. **(B)** Ophthalmic SJS means SJS/TEN with SOC.

We defined acute-stage SOC as a condition with severe conjunctivitis with pseudomembrane and epithelial defects on the ocular surface (cornea and/or conjunctiva) (11). Chronic-stage SOC was defined as a condition with sequelae such as severe dry eye, trichiasis, symblepharon, and conjunctival invasion into the cornea (Figure 1A) (10). Ophthalmologists tend to diagnose both SJS and TEN with SOC broadly as “ophthalmic SJS” (Figure 1B) (4).

Dermatologists and others reported anticonvulsants such as carbamazepine and allopurinol (a uric acid-lowering drug) as being the main SJS/TEN-inciting drugs (12), while Japanese dermatologists reported that NSAIDs and multi-ingredient cold medications were main causative drugs for SJS/TEN (13). HLA analyses have shown that a genetic predisposition plays a role in the response to disease-eliciting drugs. Carbamazepine-induced SJS/TEN manifested a very strong association with the HLA-B^*^15:02 allele in Taiwanese Han Chinese patients (14), and the HLA-A^*^31:01 allele was strongly associated with carbamazepine-induced SJS/TEN in Japanese- (15) and European patients (16), the HLA-B^*^57:01 allele was associated with carbamazepine-induced SJS/TEN in European patients (17). Allopurinol-induced SJS/TEN was strongly associated with HLA-B^*^ *58:01* in Han Chinese- (18), European ancestry- (19), and Japanese patients (20). Interestingly, not all patients with carbamazepine-induced SJS/TEN develop SOC (21). Allopurinol has been reported to elicit SJS/TEN without SOC (22).

We reported that about 80% of SJS/TEN with SOC patients seen at the Kyoto Prefectural University of Medicine developed SJS/TEN within several days after taking cold medicines (we recognized the onset of SJS/TEN when the patients had eruptions.) (8).

These included multi-ingredient cold medications and non-steroidal anti-inflammatory drugs (NSAIDs) (2, 4, 6, 8, 23). Our Brazilian collaborators found that 53% of their SJS/TEN with SOC patients had taken cold medicines (24) as had 69% of Thai patients with SJS/TEN with SOC (25), and 50% of Taiwanese patients (26). Our Korean collaborators suspected that NSAIDs and cold medicines were associated with SOC in their SJS/TEN patients (27). These observations suggest that such medicines are major causative drugs in SJS/TEN with SOC patients of different ethnicities.

This mini-review cites the results of our international collaborative efforts to identify the genetic predisposition for SJS/TEN with SOC.

## HLA Types Associated With SJS/TEN With SOC

The extreme rarity of cutaneous and ocular surface reactions to drugs led us to suspect individual susceptibility. Therefore, we entered an international collaboration to analyze the association between HLA genotypes and SJS/TEN with SOC.

### Japan

In 2007, our Japanese group first reported the HLA types associated with SJS/TEN with SOC; the ocular disease was strongly associated with HLA-A^*^02:06 [40 patients, 113 controls; odds ratio (OR) = 5.1, *p* = 0.00003] (28). Finding that about 80% of our Japanese SJS/TEN with SOC patients developed SJS/TEN after taking cold medicines to combat the common cold (8), we started to focus on cold medicine-related SJS/TEN (CM-SJS/TEN) with SOC. We reported that the ocular disease was strongly associated with HLA-A^*^02:06 [151 patients, 639 controls; (OR = 5.6, *p* = 2.7 × 10^−20^)] and significantly associated with HLA-B^*^44:03 [151 patients, 639 controls; OR = 2.0, *p* = 1.3 × 10^−3^] (2). These HLA genotypes were not associated with cold medicine-unrelated, i.e., other medicine-related SJS/TEN with SOC (2). This suggested that the associated HLA genotypes were different and depended on the causative drug(s) (2, 4, 29). Moreover, HLA-A^*^02:06 and HLA-B^*^44:03 were not associated with CM-SJS/TEN without SOC (2), suggesting that different HLA genotypes were involved in the development of SJS/TEN with- and without SOC (2).

We reported that the main causative drugs for SJS/TEN with SOC in Japanese patients were cold medicines, including multi-ingredient cold medications and NSAIDs taken to combat the common cold. As we also found that acetaminophen, present in various cold medicines, was the most frequently implicated causative drug (2, 30), we focused on acetaminophen-related SJS/TEN with SOC. Analysis of the involved HLA types revealed that HLA-A^*^02:06 was strongly associated with_-_acetaminophen-related SJS/TEN with SOC [80 patients, 113 controls; OR = 5.4, *p* = 8.0 × 10^−7^] (30).

### Korea

Together with our Korean collaborators we investigated the HLA types (HLA-A^*^02:06 and HLA-B^*^44:03) that were associated with CM-SJS/TEN with SOC in Japanese patients. We compared ours with samples from Korean patients and found that in Koreans, CM-SJS/TEN with SOC was also significantly associated with HLA-A^*^02:06 (31 patients, 90 controls; OR = 3.0, *p* = 0.018), but not with HLA-B^*^44:03 (3).

Our Korean collaborators suspected that NSAIDs and cold medicines were associated with SOC in Korean patients with SJS/TEN (27). They reported that allopurinol-induced SJS/TEN might not elicit serious acute or chronic complications of the ocular surface (22).

They then focused on Korean CM-SJS/TEN with SOC and investigated all of *HLA-class I (HLA-A, HLA-B, HLA-C*). In their patients they identified HLA-A^*^02:06 (40 patients, 120 controls; OR = 3.0, *p* = 0.0083) and HLA-C^*^03:04 (40 patients, 120 controls; OR = 3.5, *p* = 0.010) as potential positive markers for CM-SJS/TEN with SOC, and HLA-C^*^03:03 (40 patients, 120 controls; OR = 0.10, *p* = 0.0056) as a possible indicator of protection against CM-SJS/TEN with SOC in the Korean population (31).

### Brazil

Together with our Brazilian collaborators we investigated the HLA types (HLA-A^*^02:06 and HLA-B^*^44:03) that were associated with Japanese CM-SJS/TEN with SOC. Comparison of our and Brazilian samples revealed that in Brazilian CM-SJS/TEN with SOC, there was a significant association with HLA-B^*^44:03 (39 patients, 134 controls; OR = 2.7, *p* = 0.024), but not with HLA-A^*^02:06, a genotype not found in all Brazilian population (3). Interestingly, focused on European ancestry of Brazilians, the association with HLA-B^*^44:03 was stronger (15 patients, 62 controls; OR = 6.2, *p* = 0.0037) than in all Brazilians (3).

As the Brazilian collaborators found that 53% of their SJS/TEN with SOC patients had taken cold medicines before disease onset (24), they investigated the associated HLA types of CM-SJS/TEN with SOCs. Their studies suggested HLA-A^*^66:01 as a potential marker for CM-SJS/TEN with SOCs in Brazilians (39 patients, 133 controls; OR = 24.0, *p* < 0.001) of both Pardo- (19 patients, 66 controls; OR = 12.2, *p* = 0.03) and European ancestry (16 patients, 61 controls; OR = 21.2, *p* = 0.04) and that HLA-B^*^44:03 (16 patients, 61 controls; OR = 5.50, *p* = 0.01) and HLA-C^*^12:03 (16 patients, 61 controls; OR = 8.79, *p* = 0.008) might be markers only in individuals of European ancestry. Moreover, they stated that HLA-A^*^11:01 (39 patients, 133 controls; OR = 0.074, *p* = 0.008) might be a marker of resistance to CM-SJS/TEN with SOC (24).

Because Dipyrone was broadly used as cold medicine in Brazil, we also focused on dipyrone-related SJS/TEN with SOCs and found that HLA-B^*^44:03 (carrier frequency: *p* = 0.002, Pc = 0.02, OR = 8.8; gene frequency: *p* = 0.001, Pc = 0.01, OR = 7.5) and HLA-DQB1^*^04:02 (gene frequency: *p* = 0.003, Pc = 0.03, OR = 12.6) were significantly associated with cases of dipyrone-related SJS/TEN with SOCs in the Brazilian population of European ancestry, and that HLA-C^*^05:01 (carrier frequency: *p* = 0.001, Pc = 0.01, OR = 9.4; gene frequency: *p* = 0.002, Pc = 0.02, OR = 15.0) was significantly associated with cases of dipyrone-related SJS/TEN with SOCs in the Brazilian population of mixed raced ancestry (32).

### India

Together with our Indian collaborators we investigated the HLA types (HLA-A^*^02:06 and HLA-B^*^44:03) associated with Japanese CM-SJS/TEN with SOC.

In samples from Indian patients with CM-SJS/TEN with SOC there was a significant association with HLA-B^*^44:03 (20 patients, 55 controls; OR = 12.3, *p* = 1.1 × 10^−5^), but not with HLA-A^*^02:06 (3). Although the number of Indian patients was small, the association between Indian CM-SJS/TEN with SOC and HLA-B^*^44:03 was strong and significant (3).

According to Kannabiran et al. (33), Indian ophthalmologists found it difficult to obtain a detailed history of disease onset from their SJS/TEN with SOC patients and in many patients they could not identify causative drugs. HLA analysis showed that HLA-A^*^33:03 (80 patients, 50 controls; OR = 3.4, *p* = 2.7 × 10^−3^), HLA-B^*^44:03 (80 patients, 50 controls; OR = 12.2, *p* = 7.3 × 10^−9^), and HLA-C^*^07:01 (80 patients, 50 controls; OR = 6.5, *p* = 4.4 × 10^−6^) were risk alleles. HLA-B^*^57:01 (80 patients, 50 controls; OR = 0.05, *p* = 3.0 × 10^−4^) and HLA-C^*^06:02 (80 patients, 50 controls; OR = 0.1, *p* = 4.0 × 10^−4^) were protective alleles in the Indian population. Haplotypes comprised of HLA-B^*^44:03 and HLA-C^*^07:01 were strongly associated with SJS/TEN with SOC in the Indian population (80 patients, 50 controls; OR = 11.0, *p* = 1.1 × 10^−7^) (33).

### Thailand

Together with our Thai collaborators we investigated causative drugs in their SJS/TEN with SOC patients and performed HLA analysis using Thai samples. HLA-A^*^33:03 (71 patients, 159 controls; OR = 2.6, *p* = 0.0028), HLA-B^*^44:03 (71 patients, 159 controls; OR = 6.0, *p* < 0.0001), and HLA-C^*^07:01 (71 patients, 159 controls; OR = 4.9, *p* < 0.0001) exhibited a significant associations with SJS/TEN with SOC (25). Among 71 Thai SJS/TEN with SOC patients, 49 (69%) had a history of taking cold medications prior to SJS/TEN onset.

A focus on CM-SJS/TEN with SOC revealed that HLA-B^*^44:03 (49 patients, 159 controls; OR = 7.2, *p* < 0.0001) and HLA-C^*^07:01 (49 patients, 159 controls; OR = 6.1, *p* < 0.0001) were significantly associated with Thai CM-SJS/TEN with SOC. In 17 of 49 patients with CM-SJS/TEN with SOC (34.7%), a haplotype comprised of HLA-B^*^44:03 and HLA-C^*^07:01 was present. This was the case in only 11 of 159 controls (6.9 %) (OR = 7.1, *p* = 5.5 × 10^−6^), suggesting that the HLA-B^*^44:03—HLA-C^*^07:01 haplotype was a potential risk factor for CM-SJS/TEN with SOC in the Thai population (25).

In Thailand, as in the USA and UK, cold medicines, especially acetaminophen (paracetamol), are widely-used over-the-counter drugs. Elsewhere we reported that in Japan, acetaminophen is the most frequently included drug in various cold medicines (2, 30). Therefore, we focused on Japanese acetaminophen-related SJS/TEN with SOC and analyzed the HLA types (30). Together with our Thai collaborators we also investigated Thai patients with acetaminophen-related SJS/TEN with SOC and analyzed the HLA types. Jongkhajornpong et al. (34) reported a significant association with HLA-A^*^33:03 (20 patients, 60 controls; OR = 5.4, *p* = 0.0030), HLA-B^*^44:03 (20 patients, 60 controls; OR = 9.0, *p* = 0.0004), HLA-C^*^07:01 (20 patients, 60 controls; OR = 9.3, *p* = 0.0002), and the HLA-B^*^44:03—HLA-C^*^07:01 haplotype (20 patients, 60 controls; OR = 9.0, *p* < 0.001) in Thai patients with acetaminophen-related SJS/TEN with SOC, suggesting that they may have a role in the pathogenesis of SOC in acetaminophen-related SJS/TEN.

### Taiwan

Our Taiwanese collaborators found that the main causative drugs in 26 Han Chinese with SJS/TEN with SOC were cold medicines; in 13 of 26 patients with SOC, cold medicines were the causative drugs, in none of 7 patients without SOC they identified cold medications as causative (26). Their findings echoed earlier studies that implicated cold medicines in the development of SOC in 80% of Japanese SJS/TEN patients (2, 35), 53% of Brazilian patients (24), and 69% of Thai patients (25).

Together with our Taiwanese collaborators we performed HLA analysis of SJS/TEN with SOC in the Han Chinese and found that HLA-A^*^02:07 (26 patients, 98 controls; OR =3.2, *p* = 0.049) was associated with their development of the disease. Our focus on CM-SJS/TEN with SOC revealed that HLA-A^*^02:07 (13 patients, 98 controls; OR = 5.6, *p* = 0.016) was strongly associated with the development of SOC among Han Chinese CM-SJS/TEN patients (26). Single amino acid substitutions in major histocompatibility complex (MHC) class I molecules were found to play a role in distinct peptide repertoires. For example, three *HLA-A2* subtypes, i.e., HLA-A^*^02:04, HLA-A^*^02:06, and HLA-A^*^02:07, differed by only a single amino acid residue substitution; each harbored the HLA-A^*^02:01 molecule at the floor of their binding grooves. Allele-specific peptide motifs for each *HLA-A2* subtype differed substantially from the HLA-A^*^02:01 motif in the dominant anchor residues (36). Although the carrier- and gene frequency of HLA-A^*^02:06 in Japanese patients with CM-SJS/TEN with SOCs was significantly higher than in the control group, the frequency of HLA-A^*^02:07 was similar in both groups (2). We found that the expression of HLA-A^*^02:07 but not of HLA-A^*^02:06 was associated with CM-SJS/TEN with SOC in the Han Chinese patients (26).

No HLA- B^*^44:03 expression was detected in Han Chinese SJS/TEN patients or the controls (26), a finding compatible with earlier studies that showed that only 0.41–0.63% of the Taiwanese Han Chinese population harbored HLA-B^*^44:03 (37, 38). This observation suggests a genetic diversity in the pathogenesis of SJS among different ethnic groups although, because the number of samples was small, these studies must be expanded to include more samples.

### United Kingdom

Our UK collaborators found that 9 of their 28 patients with SJS/TEN with SOC (32%) had taken cold medicines (39). Together with our UK collaborators we analyzed the association of *HLA-A, HLA-B, and HLA-C* alleles with SJS/TEN in 33 patients residing in the UK (28 patients with- and 5 without SOC) and in age-matched controls. There was a statistically significant and novel negative allele association with HLA-B^*^07:02 (25 patients, 15 controls; OR = 0.16, *p* = 0.012) and with HLA-C^*^07:02 (25 patients, 15 controls; OR = 0.09, *p* = 0.030) in a sub-group of European ancestry SJS/TEN patients (both with and without SOC) but not in their controls. This finding identified these alleles as being protective (39). Interestingly, a focus on European ancestry patients with SJS/TEN with SOC revealed only the association with HLA-B^*^07:02 (23 patients, 15 controls; OR = 0.17, *p* = 0.027), but not with HLA-C^*^07:02. When the focus was directed on European ancestry patients with CM-SJS/TEN with SOC, both associations with HLA-B^*^07:02 and HLA-C^*^07:02 disappeared (39). Thus, although HLA-B^*^07:02 was associated with SJS/TEN with SOC in European ancestry, it may not be a biomarker for CM-SJS/TEN with SOC in that population. Because the number of samples was small, these studies must be expanded to include more samples.

## Discussion

A summary of our collaborative HLA analyses is shown in Table 1. It shows that HLA-B^*^44:03 was significantly associated with CM-SJS/TEN with SOC in the Japanese (2), in Brazilians, especially European ancestry Brazilians (3, 24, 32), in Indian patients (3, 33), and in Thais (25, 34). HLA-A^*^02:06 was significantly associated with CM-SJS/TEN with SOC in the Japanese (2) and in Koreans (3, 31). Ma et al. (26) suggested that HLA-A^*^02:07, differing by only a single amino acid residue substitution from HLA-A^*^02:06, might be significantly associated with CM-SJS/TEN with SOC in Taiwanese patients.

**Table 1:**
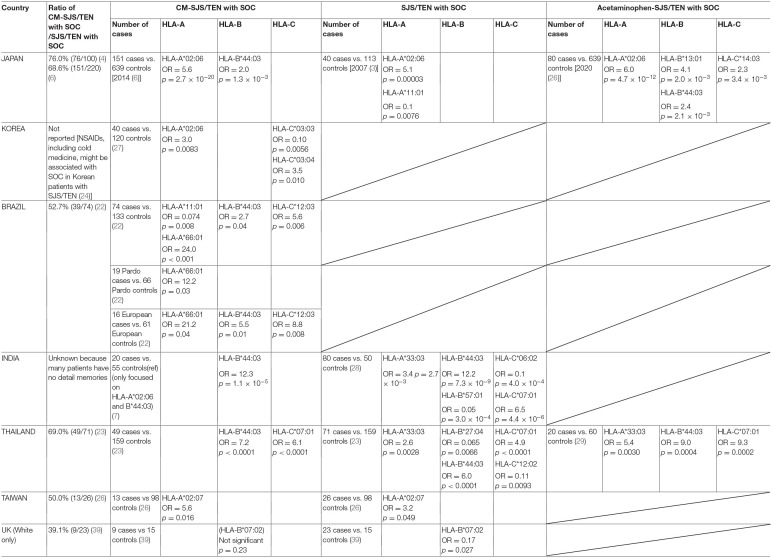
Carrier frequencies of each country.

The acetaminophen-associated HLA type might be a little different between CM-SJS/TEN with SOC in Japanese- (HLA-A^*^02:06) (30) and Thai patients (HLA-B^*^44:03—HLA-C^*^07:01 haplotype) (34). Moreover, the dipyrone-associated HLA types for CM-SJS/TEN with SOC in the Brazilian population were HLA-B^*^44:03 and HLA-DQB1^*^04:02 in European ancestry, and HLA-C^*^05:01 in mixed raced ancestry (32).

We think that a common function of cold medicines such as acetaminophen, dipyrone, and NSAIDs is highly implicated in the onset of SJS/TEN with SOC (4, 23, 30, 40).

The common function of cold medicines is the suppression of prostaglandin E_2_ (PGE_2_) production which suppress mucocutaneous inflammation. PGE_2_ acts on EP3 (PGE_2_ receptor 3) in the epidermis (41) and the mucosal epithelium (42, 43) and negatively regulates mucocutaneous inflammation. We suspect that cold medicines that include acetaminophen and dipyrone could upregulate inflammatory responses by suppressing the production of PGE_2_ which suppress mucocutaneous inflammation, that they augment abnormal immune responses, and that they elicit the induction of SJS/TEN with SOC (4, 23, 30, 40).

Besides HLA types, we investigated other SJS/TEN with SOC susceptibility genes. Our genome-wide association study revealed *IKZF1* to be a susceptibility gene for CM-SJS/TEN with SOC in Japanese-, Korean-, and Indian populations (6). It was also significantly associated with CM-SJS/TEN with SOC in Thai patients (44). Consequently, *IKZF1* may be a universal marker for CM-SJS/TEN with SOC (6, 44). Elsewhere (45) we documented that *IKZF1* regulates mucocutaneous inflammation. We reported that *IKZF1* transgenic mice developed spontaneous mucocutaneous inflammations such as ocular surface- and oral inflammation and dermatitis (45).

In the Japanese population we identified *PTGER3* as a susceptibility gene for CM-SJS/TEN with SOC (8), and we reported that HLA-A^*^02:06 and *PTGER3* polymorphisms exerted additive effects in Japanese and Korean patients with CM-SJS/TEN with SOC (OR = 10.8 and 14.2, respectively) (46).

We also suggest that in addition to microbial infections and cold medicines, the combination of multiple gene polymorphisms and their interactions contributes strongly to the onset of CM-SJS/TEN with SOC. Abnormal Innate Immunity might strongly contribute the pathology of SJS/TEN with SOC (4, 23).

Despite the genetic diversity in SJS/TEN with SOC among different ethnic groups, to prevent its onset and to reduce the incidence of blindness due to SJS/TEN, efforts must continue to identify the genetic predisposition for SJS/TEN with SOC.

## Author Contributions

MU wrote this mini review.

## Funding

This work was supported by grants-in-aid from the Ministry of Education, Culture, Sports, Science and Technology of the Japanese government, by the JSPS Core-to-Core Program, A. Advanced Research Networks, and partly supported by grants-in-aid for scientific research from the Japanese Ministry of Health, Labor, and Welfare.

## Conflict of Interest

The author declares that the research was conducted in the absence of any commercial or financial relationships that could be construed as a potential conflict of interest.

## Publisher's Note

All claims expressed in this article are solely those of the authors and do not necessarily represent those of their affiliated organizations, or those of the publisher, the editors and the reviewers. Any product that may be evaluated in this article, or claim that may be made by its manufacturer, is not guaranteed or endorsed by the publisher.

## References

[1] Sotozono C Ueta M Nakatani E Kitami A Watanabe H Sueki H. Predictive factors associated with acute ocular involvement in Stevens-Johnson syndrome and toxic epidermal necrolysis. Am J Ophthalmol. (2015) 160:228–37 e2. 10.1016/j.ajo.2015.05.002 25979679

[2] Ueta M Kaniwa N Sotozono C Tokunaga K Saito Y Sawai H. Independent strong association of HLA-A^*^02:06 and HLA-B^*^44:03 with cold medicine-related Stevens-Johnson syndrome with severe mucosal involvement. Sci Rep. (2014) 4:4862. 10.1038/srep04862 24781922 PMC5381277

[3] Ueta M Kannabiran C Wakamatsu TH Kim MK Yoon KC Seo KY. Trans-ethnic study confirmed independent associations of HLA-A^*^02:06 and HLA-B^*^44:03 with cold medicine-related Stevens-Johnson syndrome with severe ocular surface complications. Sci Rep. (2014) 4:5981. 10.1038/srep05981 25099678 PMC4124463

[4] Ueta M Kinoshita S. Ocular surface inflammation is regulated by innate immunity. Prog Retin Eye Res. (2012) 31:551–75. 10.1016/j.preteyeres.2012.05.003 22728145

[5] Ueta M Sawai H Shingaki R Kawai Y Sotozono C Kojima K. Genome-wide association study using the ethnicity-specific Japonica array: identification of new susceptibility loci for cold medicine-related Stevens-Johnson syndrome with severe ocular complications. J Hum Genet. (2017) 62:485–9. 10.1038/jhg.2016.160 28100913

[6] Ueta M Sawai H Sotozono C Hitomi Y Kaniwa N Kim MK. IKZF1, a new susceptibility gene for cold medicine-related Stevens-Johnson syndrome/toxic epidermal necrolysis with severe mucosal involvement. J Allergy Clin Immunol. (2015) 135:1538–45 e17. 10.1016/j.jaci.2014.12.1916 25672763

[7] Ueta M Sotozono C Inatomi T Kojima K Tashiro K Hamuro J. Toll-like receptor 3 gene polymorphisms in Japanese patients with Stevens-Johnson syndrome. Br J Ophthalmol. (2007) 91:962–5. 10.1136/bjo.2006.113449 17314152 PMC2266833

[8] Ueta M Sotozono C Nakano M Taniguchi T Yagi T Tokuda Y. Association between prostaglandin E receptor 3 polymorphisms and Stevens-Johnson syndrome identified by means of a genome-wide association study. J Allergy Clin Immunol. (2010) 126:1218–25 e10. 10.1016/j.jaci.2010.08.007 20947153

[9] Ueta M Sotozono C Tokunaga K Yabe T Kinoshita S. Strong association between HLA-A^*^0206 and Stevens-Johnson syndrome in the Japanese. Am J Ophthalmol. (2007) 143:367–8. 10.1016/j.ajo.2006.09.029 17258541

[10] Sotozono C Ang LP Koizumi N Higashihara H Ueta M Inatomi T. New grading system for the evaluation of chronic ocular manifestations in patients with Stevens-Johnson syndrome. Ophthalmology. (2007) 114:1294–302. 10.1016/j.ophtha.2006.10.029 17475335

[11] Sotozono C Ueta M Koizumi N Inatomi T Shirakata Y Ikezawa Z. Diagnosis and treatment of Stevens-Johnson syndrome and toxic epidermal necrolysis with ocular complications. Ophthalmology. (2009) 116:685–90. 10.1016/j.ophtha.2008.12.048 19243825

[12] Mockenhaupt M Viboud C Dunant A Naldi L Halevy S Bouwes Bavinck JN. Stevens-Johnson syndrome and toxic epidermal necrolysis: assessment of medication risks with emphasis on recently marketed drugs. the EuroSCAR-study. J Invest Dermatol. (2008) 128:35–44. 10.1038/sj.jid.5701033 17805350

[13] Yamane Y Aihara M Ikezawa Z. Analysis of Stevens-Johnson syndrome and toxic epidermal necrolysis in Japan from 2000 to 2006. Allergol Int. (2007) 56:419–25. 10.2332/allergolint.O-07-483 17713361

[14] Chung WH Hung SI Hong HS Hsih MS Yang LC Ho HC. Medical genetics: a marker for Stevens-Johnson syndrome. Nature. (2004) 428:486. 10.1038/428486a 15057820

[15] Ozeki T Mushiroda T Yowang A Takahashi A Kubo M Shirakata Y. Genome-wide association study identifies HLA-A^*^3101 allele as a genetic risk factor for carbamazepine-induced cutaneous adverse drug reactions in Japanese population. Hum Mol Genet. (2011) 20:1034–41. 10.1093/hmg/ddq537 21149285

[16] McCormack M Alfirevic A Bourgeois S Farrell JJ Kasperaviciute D Carrington M. HLA-A^*^3101 and carbamazepine-induced hypersensitivity reactions in Europeans. N Engl J Med. (2011) 364:1134–43. 10.1056/NEJMoa1013297 21428769 PMC3113609

[17] Mockenhaupt M Wang CW Hung SI Sekula P Schmidt AH Pan RY. HLA-B^*^57:01 confers genetic susceptibility to carbamazepine-induced SJS/TEN in Europeans. Allergy. (2019) 74:2227–30. 10.1111/all.13821 30972788

[18] Hung SI Chung WH Liou LB Chu CC Lin M Huang HP. HLA-B^*^5801 allele as a genetic marker for severe cutaneous adverse reactions caused by allopurinol. Proc Natl Acad Sci USA. (2005) 102:4134–9. 10.1073/pnas.0409500102 15743917 PMC554812

[19] Lonjou C Borot N Sekula P Ledger N Thomas L Halevy S. A European study of HLA-B in Stevens-Johnson syndrome and toxic epidermal necrolysis related to five high-risk drugs. Pharmacogenet Genomics. (2008) 18:99–107. 10.1097/FPC.0b013e3282f3ef9c 18192896

[20] Tohkin M Kaniwa N Saito Y Sugiyama E Kurose K Nishikawa J. A whole-genome association study of major determinants for allopurinol-related Stevens-Johnson syndrome and toxic epidermal necrolysis in Japanese patients. Pharmacogenomics J. (2013) 13:60–9. 10.1038/tpj.2011.41 21912425

[21] Kaniwa N Saito Y Aihara M Matsunaga K Tohkin M Kurose K. HLA-B locus in Japanese patients with anti-epileptics and allopurinol-related Stevens-Johnson syndrome and toxic epidermal necrolysis. Pharmacogenomics. (2008) 9:1617–22. 10.2217/14622416.9.11.1617 19018717

[22] Lee HS Ueta M Kim MK Seo KY Sotozono C Kinoshita S. Analysis of ocular manifestation and genetic association of allopurinol-induced Stevens-Johnson syndrome and toxic epidermal necrolysis in South Korea. Cornea. (2016) 35:199–204. 10.1097/ICO.0000000000000708 26655481

[23] Ueta M. Results of detailed investigations into stevens-johnson syndrome with severe ocular complications. Invest Ophthalmol Vis Sci. (2018) 59:DES183–91. 10.1167/iovs.17-23537 30481825

[24] Wakamatsu TH Ueta M Tokunaga K Okada Y Loureiro RR Costa KA. Human leukocyte antigen class I genes associated with Stevens-Johnson syndrome and severe ocular complications following use of cold medicine in a Brazilian population. JAMA Ophthalmol. (2017) 135:355–60. 10.1001/jamaophthalmol.2017.0074 28278336 PMC5470496

[25] Jongkhajornpong P Lekhanont K Pisuchpen P Chantaren P Puangsricharern V Prabhasawat P. Association between HLA-B^*^44:03-HLA-C^*^07:01 haplotype and cold medicine-related Stevens-Johnson syndrome with severe ocular complications in Thailand. Br J Ophthalmol. (2018) 102:1303–7. 10.1136/bjophthalmol-2017-311823 29706602

[26] Ma KS Chung WH Hsueh YJ Chen SY Tokunaga K Kinoshita S. Human leucocyte antigen association of patients with Stevens-Johnson syndrome/toxic epidermal necrolysis with severe ocular complications in Han Chinese. Br J Ophthalmol. (2021). 10.1136/bjophthalmol-2020-317105. [Epub ahead of print]. 33441319

[27] Lee HK Yoon KC Seo KY Ueta M Kim MK. Chronic ocular complications of Stevens-Johnson syndrome associated with causative medications in Korea. J Allergy Clin Immunol Pract. (2017) 6:700–2.e2. 10.1016/j.jaip.2017.09.001 28988785

[28] Ueta M Sotozono C Inatomi T Kojima K Hamuro J Kinoshita S. Association of IL4R polymorphisms with Stevens-Johnson syndrome. J Allergy Clin Immunol. (2007) 120:1457–9. 10.1016/j.jaci.2007.07.048 17900677

[29] Ueta M. Genetic predisposition to stevens-johnson syndrome with severe ocular surface complications. Cornea. (2015) 34(Suppl. 11):S158–65. 10.1097/ICO.0000000000000605 26448174

[30] Ueta M Nakamura R Saito Y Tokunaga K Sotozono C Yabe T. Association of HLA class I and II gene polymorphisms with acetaminophen-related Stevens-Johnson syndrome with severe ocular complications in Japanese individuals. Hum Genome Var. (2019) 6:50. 10.1038/s41439-019-0082-6 31666976 PMC6817890

[31] Jun I Rim JH Kim MK Yoon KC Joo CK Kinoshita S. Association of human antigen class I genes with cold medicine-related Stevens-Johnson syndrome with severe ocular complications in a Korean population. Br J Ophthalmol. (2019) 103:573–6. 10.1136/bjophthalmol-2018-313263 30705045

[32] Wakamatsu TH Ueta M Inoue C Costa KA Sakano LY Sallum JMF. Human leukocyte antigen class I and II genes associated with dipyrone-related Stevens-Johnson syndrome and severe ocular complications in a Brazilian population. Ocul Surf. (2021) 20:173–5. 10.1016/j.jtos.2021.02.008 33617977

[33] Kannabiran C Ueta M Sangwan V Rathi V Basu S Tokunaga K. Association of human leukocyte antigen class 1 genes with Stevens Johnson syndrome with severe ocular complications in an Indian population. Sci Rep. (2017) 7:15960. 10.1038/s41598-017-15965-7 29162886 PMC5698496

[34] Jongkhajornpong P Ueta M Lekhanont K Puangsricharern V Prabhasawat P Chantaren P. Association of HLA polymorphisms and acetaminophen-related Steven-Johnson syndrome with severe ocular complications in Thai population. Br J Ophthalmol. (2020). 10.1136/bjophthalmol-2020-317315. [Epub ahead of print]. 33229345

[35] Ueta M. Cold medicine-related Stevens-Johnson syndrome/toxic epidermal necrolysis with severe ocular complications-phenotypes and genetic predispositions. Taiwan J Ophthalmol. (2016) 6:108–18. 10.1016/j.tjo.2016.06.001 29018724 PMC5525617

[36] Sudo T Kamikawaji N Kimura A Date Y Savoie CJ Nakashima H. Differences in MHC class I self peptide repertoires among HLA-A2 subtypes. J Immunol. (1995) 155:4749–56. 7594476

[37] Harr T French LE. Stevens-Johnson syndrome and toxic epidermal necrolysis. Chem Immunol Allergy. (2012) 97:149–66. 10.1159/000335627 22613860

[38] Wen SH Lai MJ Yang KL. Human leukocyte antigen-A, -B, and -DRB1 haplotypes of cord blood units in the Tzu Chi Taiwan cord blood bank. Hum Immunol. (2008) 69:430–6. 10.1016/j.humimm.2008.05.012 18582515

[39] Butt GF Hassan A Wallace GR Kinoshita S Ahmad S Ueta M. Human leukocyte antigen B^*^0702 is protective against ocular Stevens-Johnson syndrome/toxic epidermal necrolysis in the UK population. Sci Rep. (2021) 11:2928. 10.1038/s41598-021-82400-3 33536518 PMC7859395

[40] Ueta M. Stevens-Johnson syndrome/toxic epidermal necrolysis with severe ocular complications. Expert Rev Clin Immunol. (2020) 16:285–91. 10.1080/1744666X.2020.1729128 32045311

[41] Honda T Matsuoka T Ueta M Kabashima K Miyachi Y Narumiya S. Prostaglandin E(2)-EP(3) signaling suppresses skin inflammation in murine contact hypersensitivity. J Allergy Clin Immunol. (2009) 124:809–18 e2. 10.1016/j.jaci.2009.04.029 19541354

[42] Kunikata T Yamane H Segi E Matsuoka T Sugimoto Y Tanaka S. Suppression of allergic inflammation by the prostaglandin E receptor subtype EP3. Nat Immunol. (2005) 6:524–31. 10.1038/ni1188 15806106

[43] Ueta M Matsuoka T Narumiya S Kinoshita S. Prostaglandin E receptor subtype EP3 in conjunctival epithelium regulates late-phase reaction of experimental allergic conjunctivitis. J Allergy Clin Immunol. (2009) 123:466–71. 10.1016/j.jaci.2008.09.044 18996575

[44] Chantaren P Jongkhajornpong P Ueta M Puangsricharern V Lekhanont K Pisuchpen P. Association of IKZF1 SNPs in cold medicine-related Stevens-Johnson syndrome in Thailand. Clin Transl Allergy. (2019) 9:61. 10.1186/s13601-019-0300-9 31768251 PMC6873726

[45] Ueta M Hamuro J Nishigaki H Nakamura N Shinomiya K Mizushima K. Mucocutaneous inflammation in the Ikaros Family Zinc Finger 1-keratin 5-specific transgenic mice. Allergy. (2018) 73:395–404. 10.1111/all.13308 28914974

[46] Ueta M Tokunaga K Sotozono C Sawai H Yoon KC Kim MK. HLA-A^*^02:06 and PTGER3 polymorphism exerts additive effects in cold medicine-related Stevens-Johnson syndrome with severe ocular complications. Hum Genome Variat. (2015) 2:15023. 10.1038/hgv.2015.23 27081535 PMC4785539

